# Supplementary material for: Targeting light-gated chloride channels to neuronal somatodendritic domain reduces their excitatory effect in the axon
Source: eLife. 2018 Aug 9;7:e38506. doi: 10.7554/eLife.38506 (PMC6130974; doi:10.7554/eLife.38506)
Supplement: Supplementary file 1. — The statistical tests, numbers of replicates and mice, and P values were reported for all figures and experiments. [file elife-38506-supp1.docx]

| **Figure** | **Statistical Test** | ***n***  **(neuron or ROI)** | ***N***  **(mouse)** | ***P* value** |
| --- | --- | --- | --- | --- |
| 1C |  | 5 | 3 |  |
| 1D |  | 2 | 2 |  |
| 1F | Wilcoxan matched-pairs signed rank | 17 | 7 | < 0.0001 |
| 1G | Paired *t* test | 8 | 4 | 0.0223 |
| 1J | Paired *t* test | 7 | 4 | 0.0247 |
| 1K | Wilcoxan matched-pairs signed rank | 10 | 4 | 0.0020 |
| 1-supplement 1 |  | 3 | 2 |  |
| 1-supplement 2B |  | 12 | 5 |  |
| 1-supplement 2D |  | 14 | 2 |  |
| 1-supplement 3C |  | 6 | 1 |  |
| 1-supplement 3D |  | 6 | 1 |  |
| 1-supplement 3H |  | 7 | 1 |  |
| 1-supplement 3I |  | 7 | 1 |  |
| 2B | Wilcoxan matched-pairs signed rank | 10 | 5 | 0.0020 |
| 2D | Wilcoxan matched-pairs signed rank | 8 | 2 | 0.9453 |
| 2F | Wilcoxan matched-pairs signed rank | 9 | 3 | 0.0039 |
| 2H | Paired *t* test | 6 | 2 | 0.0085 |
| 2J | Wilcoxan matched-pairs signed rank | 8 | 2 | 0.0078 |
| 2-supplement 1C | Linear regression |  |  |  |
| 2-supplement 1D | Paired *t* test | 8 | 3 | 0.4044 |
| 2-supplement 2B | Wilcoxan matched-pairs signed rank | 12 | 7 | 0.0342 |
| 2-supplement 2E | Wilcoxan matched-pairs signed rank | 12 | 3 | 0.0005 |
| 2-supplement 3B | Wilcoxan matched-pairs signed rank  EPSCs  Paired *t* test  Photocurrent | 6 | 2 | 0.0312  0.2651 |
| 2-supplement 3D | Paired *t* test  EPSCs  Photocurrent | 3 | 1 | 0.0254  0.7496 |
| 3B | Wilcoxan matched-pairs signed rank | 15 | 3 | < 0.0001 |
| 3D | Repeated measures one-way ANOVA with Greenhouse-Geisser correction and Tukey multiple comparisons test with multiplicity adjusted *P* value  TTX vs Baseline  4-AP + TEA vs Baseline  TTX vs TTX + 4-AP + TEA | 10 | 2 | 0.0094  0.0179  0.0326  0.0004 |
| 3F | Repeated Measures one-way ANOVA with Greenhouse-Geisser correction with Tukey multiple comparisons test with multiplicity adjusted *P* value  TTX vs Baseline  4-AP+TEA vs Baseline  TTX vs TTX+4AP+TEA | 9 | 2 | 0.0038  0.0061  0.0163  0.0076 |
| 3H | Loose patch recording  Whole-cell recording | 88  56 | 15  12 |  |
| 3J | Loose patch recording  Whole-cell recording | 11  17 | 2  4 |  |
| 3-supplement 1C | Wilcoxan matched-pairs signed rank | 9 | 3 | 0.0039 |
| 4D | Kruskal-Wallis with Dunn’s multiple comparisons test (vs WT) with multiplicity adjusted *P* value  WT  MBD  Nlgn1C  Kv4.2LL  KA2N  TlcnC  Kv2.1C  Kv2.1-TlcnC  Kv2.1-linker-TlcnC | 24  6  6  6  11  6  18  6  18 | 4  1  1  1  2  1  3  1  3 | < 0.0001  0.7997  > 0.99  > 0.99  0.5372  0.4272  < 0.0001  0.0011  < 0.0001 |
| 4E | Ordinary one-way ANOVA with Dunnet’s multiple comparison test with multiplicity adjusted *P* value  WT  Kv2.1C  Kv2.1C-linker-TlcnC | 12  15  11 | 4  4  4 | 0.0023  0.0043  0.0039 |
| 5C | *t* test with Welch’s correction  WT vs Kv2.1C  Mann-Whitney test  WT vs Kv2.1C-linker-TlcnC | 13 vs 18  22 vs 34 | 3 vs 4  6 vs 7 | < 0.0001  < 0.0001 |
| 5F | Mann-Whitney test  WT vs Kv2.1C  WT vs Kv2.1C-linker-TlcnC | 17 vs 21  37 vs 43 | 3 vs 4  6 vs 7 | 0.0026  0.0019 |
| 5G (left) | Mann-Whitney test  WT vs Kv2.1C  WT vs Kv2.1C-linker-TlcnC | 18 vs 21  35 vs 44 | 3 vs 4  6 vs 7 | 0.0028  < 0.0001 |
| 5G (right) | Mann-Whitney test  WT vs Kv2.1C  WT vs Kv2.1C-linker-TlcnC | 18 vs 21  43 vs 46 | 3 vs 4  6 vs 7 | 0.0258  < 0.0001 |
| 5I | Mann-Whitney test  WT vs Kv2.1C  WT vs Kv2.1C-linker-TlcnC | 15 vs 20  25 vs 21 | 3 vs 4  5 vs 5 | 0.0010  0.0002 |
| 5-supplement 1C | Mann-Whitney test  WT vs Kv2.1C  WT vs Kv2.1C-linker-TlcnC | 13 vs 19  23 vs 32 | 3 vs 4  6 vs 7 | < 0.0001  < 0.0001 |
| 5-supplement 1D | Mann-Whitney test  WT vs Kv2.1C  WT vs Kv2.1C-linker-TlcnC | 17 vs 21  35 vs 39 | 3 vs 4  6 vs 6 | 0.0023  0.0002 |
| 5-supplement 1E | WT  Kv2.1C | 12  17 | 3  4 |  |
| 5-supplement 1F | WT  Kv2.1C | 17  21 | 3  4 |  |
| 5-supplement 1G | WT  Kv2.1C-linker-TlcnC | 19  29 | 6  7 |  |
| 5-supplement 1H | WT  Kv2.1C-linker-TlcnC | 31  38 | 4  6 |  |
| 5-supplement 1I | Kv2.1C-linker-TlcnC | 3 | 3 |  |
| iC++ vs iChloC photocurrent  (no figure) | *t* test with Welch’s correction  iChloC  iC++ | 7  15 | 2  5 | < 0.0001 |
| iCholC-induced IPSC  (no figure) |  | 10 | 2 |  |
| GtACR1-induced EPSC  (no figure) |  | 11 | 3 |  |
